# Supplementary material for: Clr4SUV39H1 ubiquitination and non-coding RNA mediate transcriptional silencing of heterochromatin via Swi6 phase separation
Source: Nat Commun. 2024 Oct 30;15:9384. doi: 10.1038/s41467-024-53417-9 (PMC11526040; doi:10.1038/s41467-024-53417-9)
Supplement: Supplementary file 1 — Supplementary Information [file 41467_2024_53417_MOESM1_ESM.pdf]

a

```
                                                                   48
spUbc4   MALKRINRELADLGKDPPSSCSAGPVGDDLFHWQATIMGPADSPYAGGVFFLSIHFPTDY 60
mUBE2D2  MALKRIHKELNDLARDPPAQCSAGPVGDDMFHWQATIMGPNDSPYQGGVFFLTIHFPTDY 60
mUBE2D3  MALKRINKELSDLARDPPAQCSAGPVGDDMFHWQATIMGPNDSPYQGGVFFLTIHFPTDY 60
         ******::** **.:***:.*********:********** **** ******:*******

spUbc4   PFKPPKVNFTTRIYHPNINSNGSICLDILRDQWSPALTISKVLLSICSLLTDPNPDDPLV 120
mUBE2D2  PFKPPKVAFTTRIYHPNINSNGSICLDILRSQWSPALTISKVLLSICSLLCDPNPDDPLV 120
mUBE2D3  PFKPPKVAFTTRIYHPNINSNGSICLDILRSQWSPALTISKVLLSICSLLCDPNPDDPLV 120
         ******* **********************.******************* *********

                          137
spUbc4   PEIAHVYKTDRSRYELSAREWTRKYAI 147
mUBE2D2  PEIARIYKTDREKYNRIAREWTQKYAM 147
mUBE2D3  PEIARIYKTDRDKYNRISREWTQKYAM 147
         ****::*****.:*:  :****:***:

Red(*)   : Identical
Blue(:)  : Strongly similar
Green(.) : Weakly similar
Black( ) : None
```

b

	spUbc4	mUBE2D2	mUBE2D3
spUbc4	100 (100)	84.4 (95.2)	84.4 (95.9)
mUBE2D2	84.4 (95.2)	100 (100)	97.3 (100)
mUBE2D3	84.4 (95.9)	97.3 (100)	100 (100)

Identity (Similarity), %

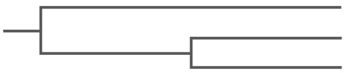

c

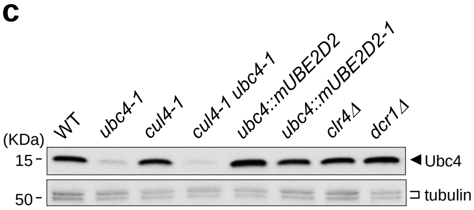

Supplementary Fig. 1│ Comparison of Ubc4 with mouse homologs.

a Left, Amino acid sequence comparison between Ubc4 with its mouse homologs using Clustal Omega. Glycine 48 of Ubc4 is conserved but Serine 137, which is important for Ubc4 stability (see below), is not conserved.

b The summary of amino acid sequence comparison between *S. poombe* Ubc4 with mouse UBE2D2 and UBE2D3. The comparison of Ubc4 with mUBE2D3 is highlighted with red. Bottom, cladogram for the relationship of these proteins.

c Ubc4 protein was greatly reduced in *ubc4-G48D* (*ubc4-1)* mutant. For WB analyses of Ubc4 protein in the indicated strains, antibody for mUBE2D3 was used to detect Ubc4. WB of tubulin was used as loading control. Molecular weight markers are shown and uncropped images are provided in a Source Data file.

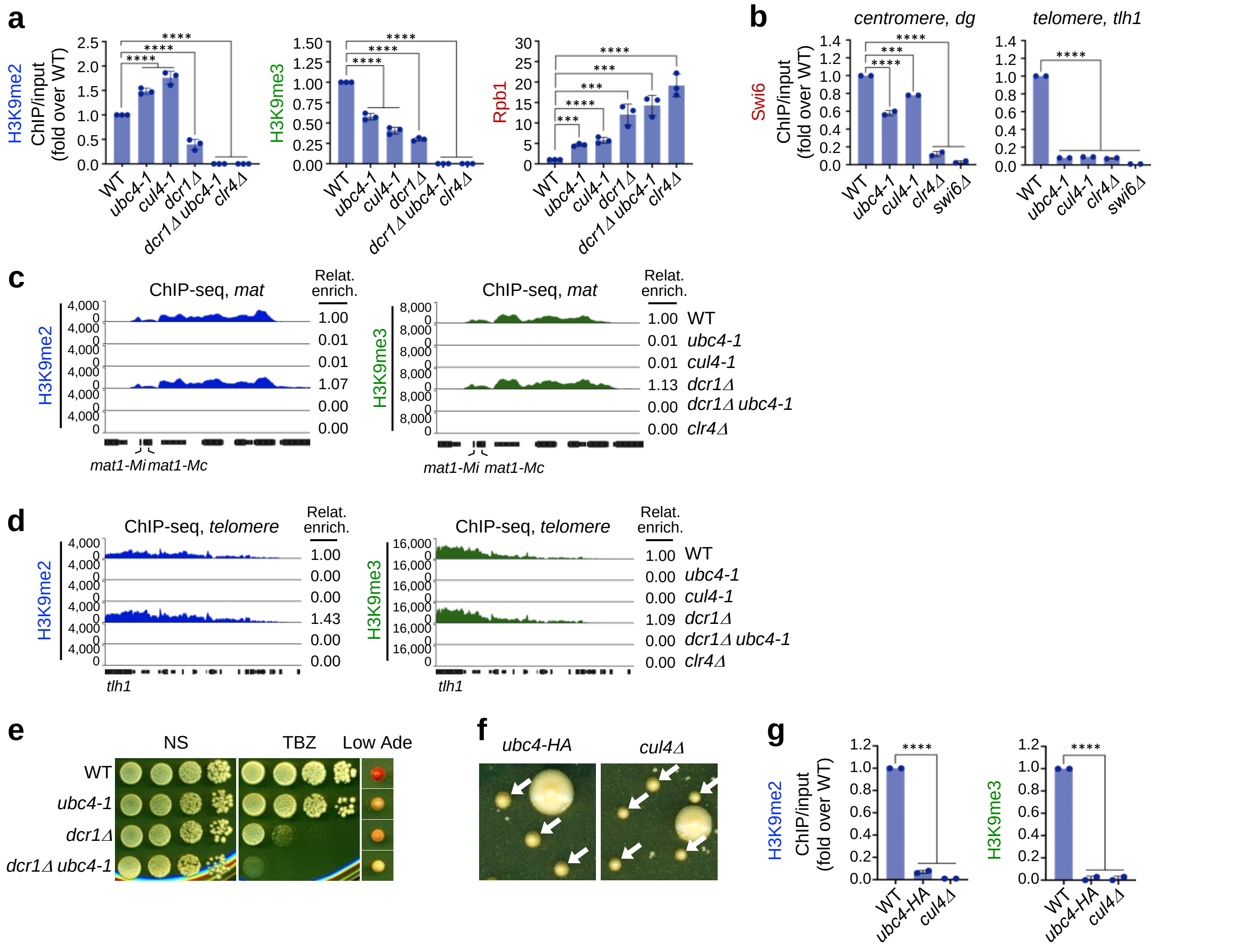

Supplementary Fig. 2 | Ubc4 and Cul4 regulate heterochromatin silencing.

a ChIP-qPCR assays showing enrichment of H3K9me2 (left), H3K9me3 (middle) and Rpb1 (right) at centromeric *dh* locus in the indicated strains. Error bars, S.D. (n = 3). Data are presented as mean ± SD (n = 3). *P* values are from one-way ANOVA test (Dunnett's multiple comparisons test). *P* < 0.0001 for all mutants (left and middle). *P* = 0.0002 for *ubc4-1*, *P* < 0.0001 for *cul4-1*, *P* = 0.0008 for *dcr1Δ*, *P* = 0.0003 for *dcr1Δ ubc4-1* and *P* < 0.0001 for *clr4Δ* (right).

b ChIP-qPCR assays showing enrichment of HP1 protein, Swi6 at centromeric *dg* locus (left) and telomere 1 left region (right) in the indicated strains. Error bars, S.D. (n = 2). Data are presented as mean ± SD (n = 3). *P* values are from one-way ANOVA test (Dunnett's multiple comparisons test). *P* < 0.0001 for *ubc4-1*, *P* = 0.0003 for *cul4-1* and *P* < 0.0001 for *clr4Δ* and *swi6Δ* (left). *P* < 0.0001 for all mutants (right).

c and **d** ChIP-seq reads of H3K9me2 and H3K9me3 mapped to mating type locus (**b**) and telomere (**c**) in the indicated strains.

e Assays for silencing of centromeric *ade6⁺* on Low Ade medium and for chromosome segregation by viability on 12.5 μg/ml TBZ medium in the indicated strains.

f *ubc4* mutants tagged with triple HA at carboxyl-terminus (*ubc4-HA*) and *cul4* deletion mutants (*cul4Δ*) are viable but have a severe growth defect (arrows). Large colonies are WT controls.

g ChIP-qPCR assays showing enrichment of H3K9me2 and H3K9me3 at centromeric *dg* repeat in the indicated strains. Error bars, S.D. (n = 2). Data are presented as mean ± SD (n = 2). *P* values are from one-way ANOVA test (Dunnett's multiple comparisons test). *P* < 0.0001 for all mutants (left and right).

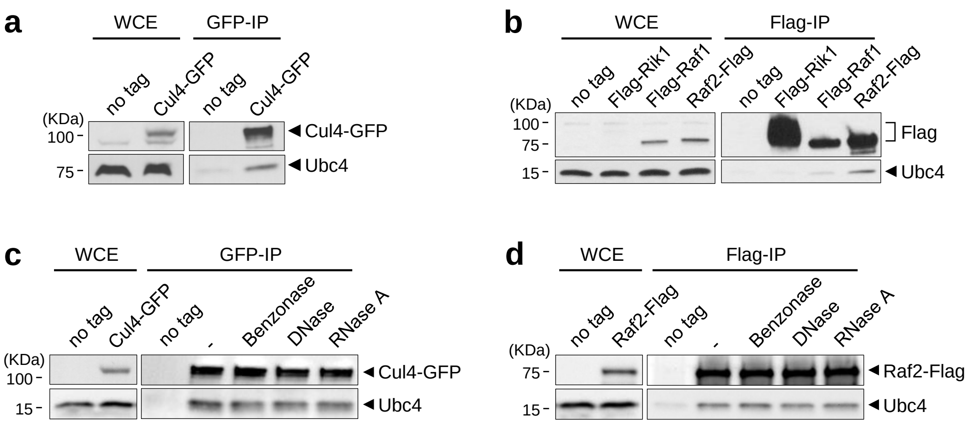

Supplementary Fig. 3 │ Ubc4 and Cul4 form Ubc4-CLRC complex independently of nucleases.
a and **b** Co-immunoprecipitation (Co-IP) of Cul4-GFP (**a**) and Flag-Rik1, Flag-Raf1 and Raf2-Flag (**b**) with Ubc4. Molecular weight markers are shown and uncropped images are provided in a Source Data file.
c and **d** Co-IP of Cul4-GFP (**c**) and Raf2-Flag (**d**) with Ubc4 in the absence and presence of Benzonase (1.25 U/μl), TURBO DNase (0.1 U/μl) and RNase A (0.5 μg/μl). Molecular weight markers are shown and uncropped images are provided in a Source Data file.

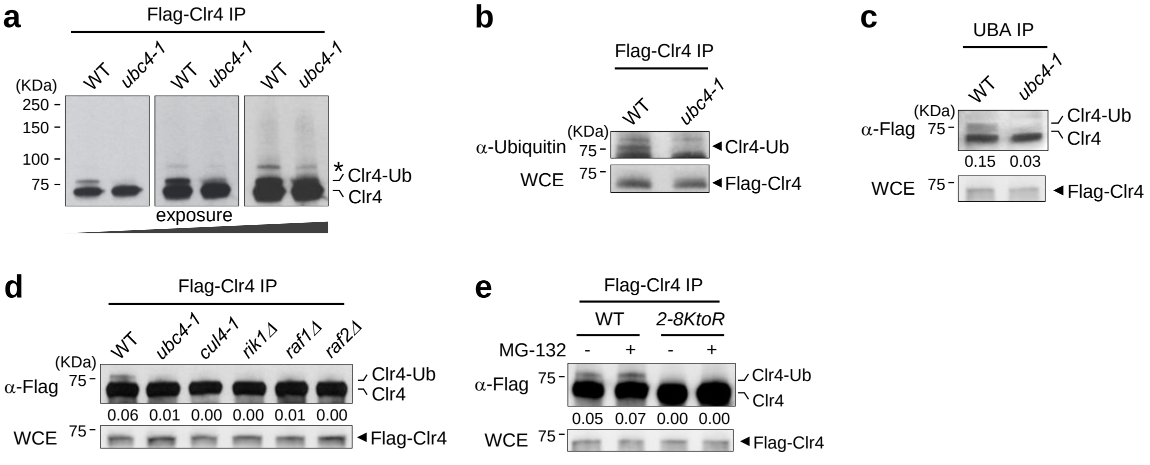

Supplementary Fig. 4│ Mono-ubiquitination of Clr4.

a Extended exposure of WB analysis for Flag-Clr4 IP from WT and *ubc4-1* mutant cells (Fig. 1B). Asterisk indicates possible di- ubiquitination which is also largely dependent on Ubc4. Molecular weight markers are shown and uncropped images are provided in a Source Data file.

b Flag-Clr4 IP and WB analysis using ubiquitin antibody. Molecular weight markers are shown and uncropped images are provided in a Source Data file.

c WB analyses Flag-Clr4 enrichment by ubiquitination affinity beads (UBA IP). Molecular weight markers are shown and uncropped images are provided in a Source Data file.

d WB analyses of immunoprecipitated Flag-Clr4 (Flag-Clr4 IP) in the indicated strains. Molecular weight markers are shown and uncropped images are provided in a Source Data file.

e Proteasome-independent Clr4 ubiquitination. WB analyses of Flag-Clr4 IP with or without treatment of proteasome inhibitor MG-132 in the indicated strains. Molecular weight markers (KDa) are shown and uncropped images are provided in a Source Data file.

a

Sequence	A.A.	Prob	Modifications
(R)NGAVKLYR(I)	K25	**99%**	GG (+114)
(R)LKGSNSDSDSPHHASNPHPNSR(Q)	K65	**99%**	GG (+114)
(K)KVFSSQTTKR(Q)	K114	**96%**	GG (+114)
(R)KQSTALTTNDTSIILDDSLHTNSKK(L)	K151	**96%**	GG (+114)
(K)TRNEVKEESQKR(E)	K160	85%	GG (+114)
(R)ELVSNSIKEATSPK(T)	K174	84%	GG (+114)
(R)ELVSNSIKEATSPKTSSILTKPR(N)	K180	**100%**	GG (+114)
(K)TSSILTKPR(N)	K187	**97%**	GG (+114)
(R)TLPLEIFKTK(E)	K336	**99%**	GG (+114)

c

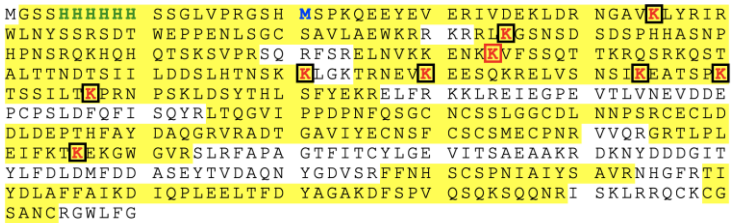

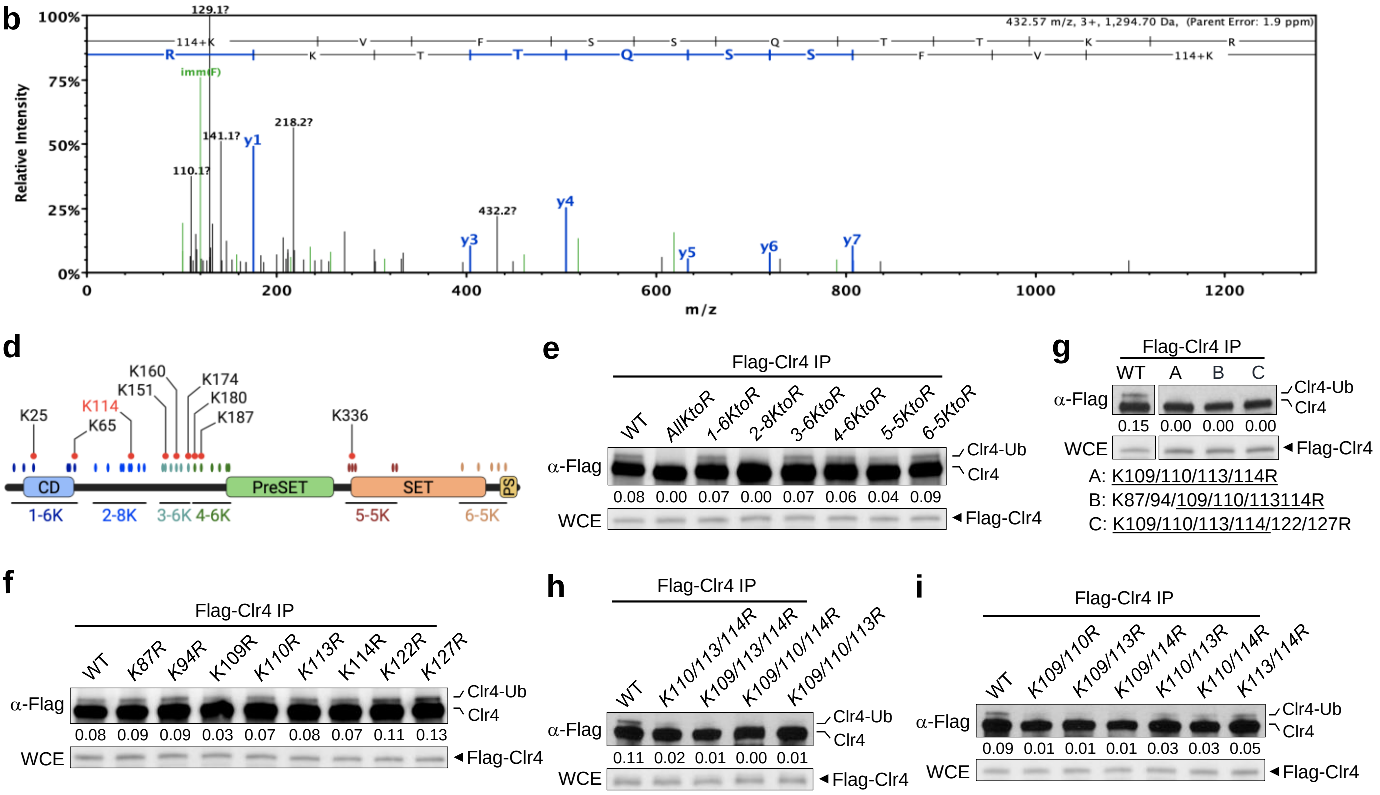

Supplementary Fig. 5 | Characterization of ubiquitination sites of Clr4 by Mass Spec and genetic analyses.
a 6xHis-Clr4 purified from *E. coli* cells were *in vitro* ubiquitinated and analyzed by Mass Spectrometry. Di-glycine (GG) remnants after trypsin digestion indicate possible ubiquitination sites. Prob., probability. K114 is highlighted.
b MS/MS spectrum of the peptide containing ubiquitinated K114. The observed y ions are shown. Predicted amino acids sequences are across the top.
c Amino acid sequence of 6xHis-Clr4 protein. Sequence coverage is highlighted in yellow. 6xHis (green) and translation start site (blue) are shown. Possible ubiquitination sites (red) are highlighted with black boxes and K114 is highlighted with red box.
d Schematic diagram of domain structure of Clr4 and ubiquitination sites characterized by Mass Spectrometry analysis. K114 is highlighted with red.
e-i WB analyses of Flag-Clr4 IP from indicated strains. Molecular weight markers and uncropped images are provided in a Source Data file.

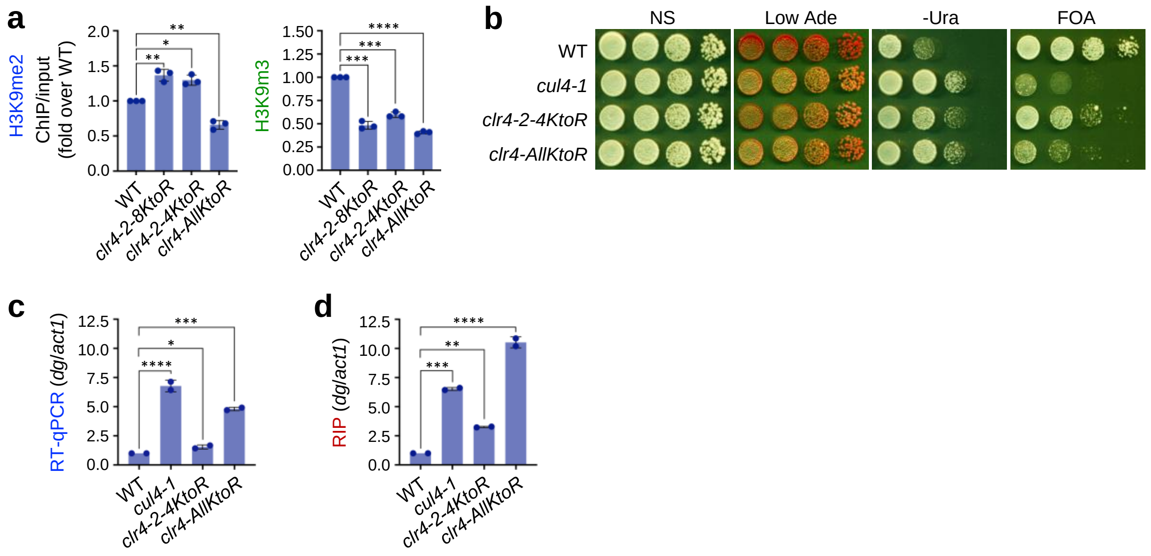

Supplementary Fig. 6 | Heterochromatin silencing defects in *clr4-2-8KtoR*, *clr4-2-4KtoR* and *clr4-AllKtoR* mutants.

a ChIP-qPCR assays showing enrichment of H3K9me2 and H3K9me3 at centromeric *dh* repeat in the indicated strains. Data are presented as mean ± SD (n = 3). *P* values are from one-way ANOVA test (Dunnett's multiple comparisons test). $P = 0.0025$ for *clr4-2-8KtoR*, $P = 0.0233$ for *clr4-2-4KtoR* and $P = 0.0039$ for *clr4-AllKtoR* (left). $P = 0.0002$ for *clr4-2-8KtoR*, $P = 0.0003$ for *clr4-2-4KtoR* and $P < 0.0001$ for *clr4-AllKtoR* (right).

b Assays for silencing of centromeric *ade6*$^{+}$ and *ura4*$^{+}$ on Low Ade, -Ura and FOA media in the indicated strains.

c RT-qPCR analysis for centromeric *dg* repeat (normalized to *act1*). Data are presented as mean ± SD (n = 2). *P* values are from one-way ANOVA test (Dunnett's multiple comparisons test). $P < 0.0001$ for *cul4-1*, $P = 0.0433$ for *clr4-2-4KtoR* and $P = 0.0004$ for *clr4-AllKtoR.*

d RNA immunoprecipitation (RNA IP) and qPCR of Flag-Clr4 to centromeric *dg* and *act1* transcript in the indicated strains. Data are presented as mean ± SD (n = 2). *P* values are from one-way ANOVA test (Dunnett's multiple comparisons test). $P = 0.0001$ for *cul4-1*, $P = 0.0063$ for *clr4-2-4KtoR* and $P < 0.0001$ for *clr4-AllKtoR.*

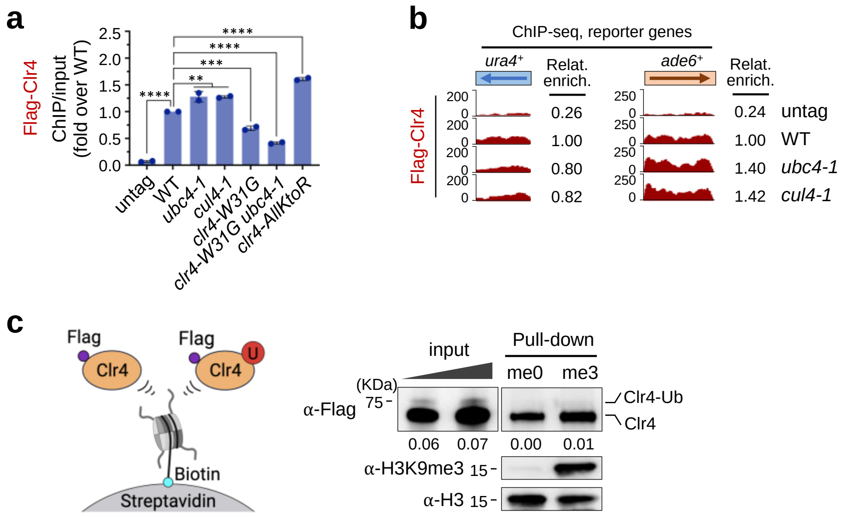

Supplementary Fig. 7 | Clr4 mono-ubiquitination induces its dissociation from chromatin and from centromeric ncRNA.

a ChIP-qPCR assays showing enrichment of Flag-Clr4 at centromeric *dh* repeat in the indicated strains. Data are presented as mean ± SD (n = 2). *P* values are from one-way ANOVA test (Dunnett's multiple comparisons test). *P* < 0.0001 for untag, *P* = 0.0017 for *ubc4-1*, *P* = 0.0018 for *cul4-1*, *P* = 0.0009 for *clr4-W31G* and *P* < 0.0001 for *clr4-W31G ubc4-1* and *clr4Δ*.

b ChIP-seq reads of Flag-Clr4 mapped to *ura4*⁺ and *ade6*⁺ reporter genes in indicated strains.

c Binding assay of Flag-Clr4 protein purified from *S. pombe* cells with nucleosomes with unmodified histone H3 (me0) or H3K9me3 histone (me3) anchored to streptavidin beads. Input and Flag-Clr4 proteins from pull-down were analyzed by WB using antibodies as shown. Schematic diagram of binding assay (left). Molecular weight markers are shown and uncropped images are provided in a Source Data file.

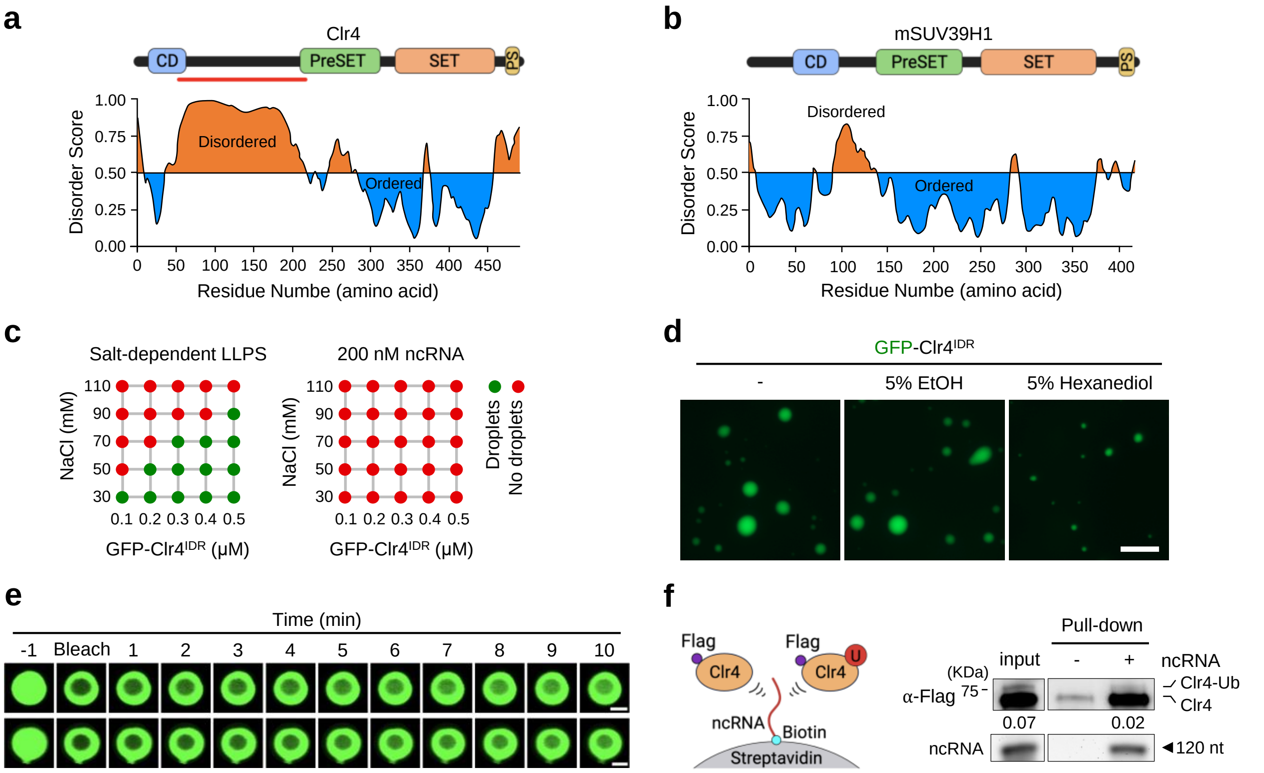

Supplementary Fig. 8 | Phase separation of Clr4.

a Schematic diagram of domain structure of Clr4 protein. Disorder prediction shows that the hinge region between the chromodomain (CD) and PreSET domain of Clr4 is highly disordered. Red bar shows the location of amino acids which is used for GFP-Clr4^{IDR} fusion (51-220 amino acids).

b Schematic diagram of domain structure and disorder prediction (PONDR) of mSUV39H1 protein.

c Summary of LLPS behaviors of Clr4^{IDR} with different concentrations of salt (NaCl, left). Summary of LLPS behaviors of Clr4^{IDR} with different concentrations of salt in the presence of 200 nM centromeric ncRNA (right).

d Representative images of phase-separated liquid droplets of GFP-Clr4^{IDR} after treatment with 5% Ethanol (EtOH) or 5% Hexanediol. Scale bar, 5 μm.

e FRAP of GFP-Clr4^{Full}. Representative phase-separated liquid droplests were monitored as shown. Photo bleach was applied around time 0 min. Scale bar, 2 μm.

f Flag-Clr4 protein purified from *S. pombe* cells were incubated with centromeric ncRNA anchored to streptavidin beads. Input and bound Flag-Clr4 proteins from pull-down were analyzed by WB. ncRNA from input and pull-down was stained with SYBR Gold. Schematic diagram (left). Molecular weight marker is shown and uncropped images are provided in a Source Data file.

Supplementary Fig. 9

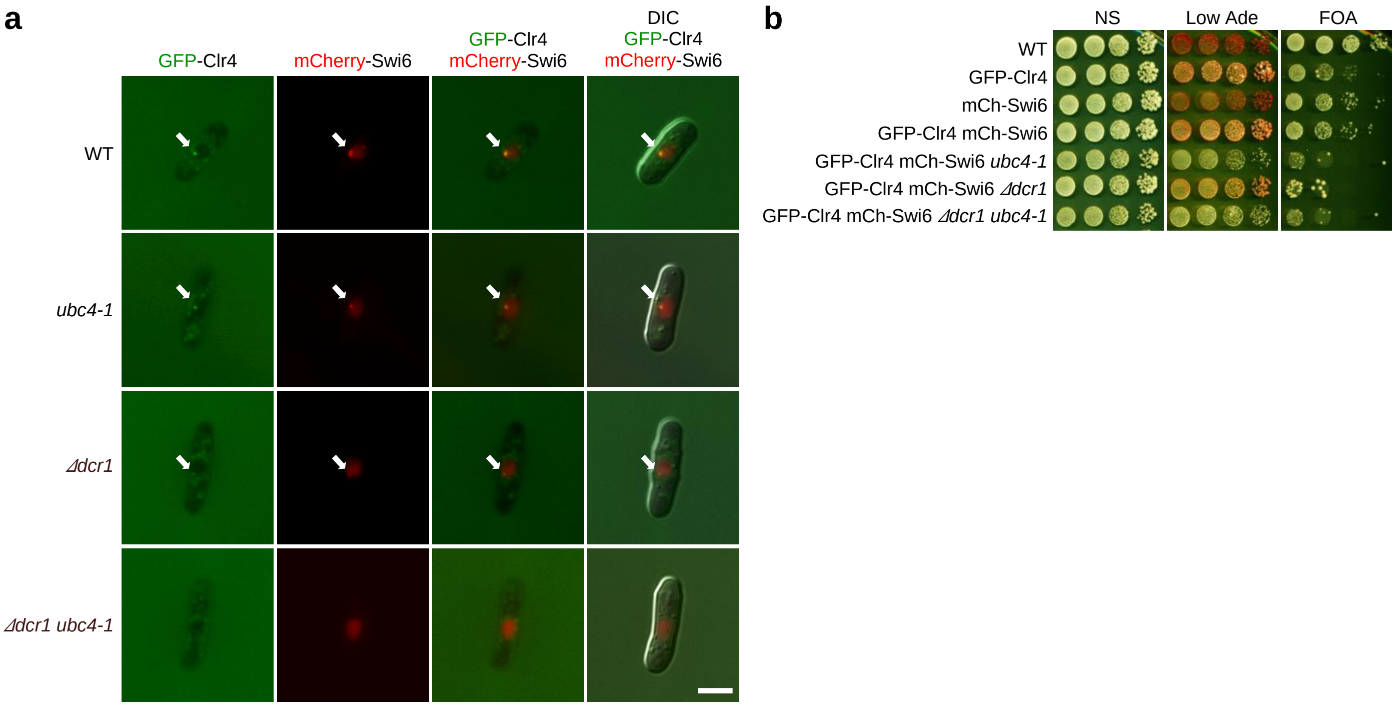

Supplementary Fig. 9 | Co-localization of GFP-Clr4 and mCherry-Swi6.
a Live cell imaging showing condensed GFP-Clr4 and mCherry-Swi6 heterochromatic foci in indicated strains. Co-localization of GFP-Clr4 and mCherry-Swi6 condensates were seen clearly in WT and with reduced intensity in *ubc4-1* and *dcr1Δ* mutants. Both GFP-Clr4 and mCherry-Swi6 condensates are completely lost in *dcr1Δ ubc4-1* mutant. Scale bar, 2.5 μm.
b Assays for silencing of centromeric *ade6*$^+$ and *ura4*$^+$ on Low Ade and FOA media in the indicated strains.

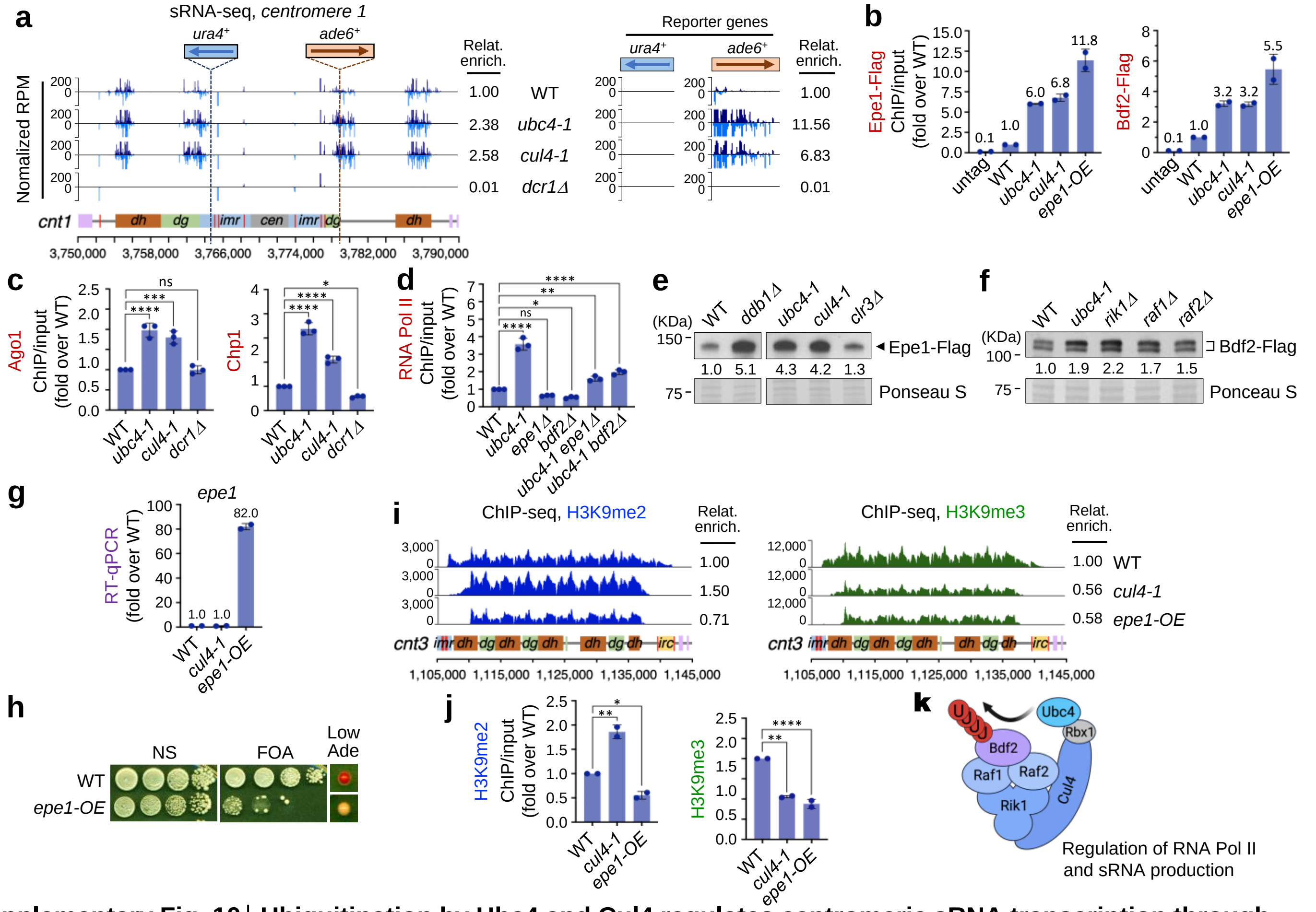

Supplementary Fig. 10 | Ubiquitination by Ubc4 and Cul4 regulates centromeric sRNA transcription through Epe1 and Bdf2[BRD4].

a sRNA-seq reads mapped to chromosome 1 centromere. The *ura4*⁺ reporter gene is inserted in *imr* with no sRNA production whereas *ade6*⁺ is located in *otr* where sRNA are produced. Increased sRNA production was observed at the *ade6*⁺ reporter gene, but not at the *ura4*⁺ reporter gene, in *ubc4-1* and *cul4-1* mutants.

b ChIP-qPCR assays for enrichment of Epe1-Flag and Bdf2-Flag at centromeric *dh* repeat in the indicated strains. Data are presented as mean ± SD (n = 2).

c ChIP-qPCR assays for enrichment of Ago1 and Chp1 at centromeric *dh* repeat in the indicated strains. Data are presented as mean ± SD (n = 3). *P* values are from one-way ANOVA test (Dunnett's multiple comparisons test). *P* < 0.0001 for *ubc4-1* and *P* = 0.0002 for *cul4-1* (left). *P* < 0.0001 for *ubc4-1* and *cul4-1* and *P* = 0.0206 for *dcr1Δ* (right).

d ChIP-qPCR assays for enrichment of RNA Pol II (Rpb1) at centromeric *dg* repeat in the indicated strains. Data are presented as mean ± SD (n = 3). *P* values are from one-way ANOVA test (Dunnett's multiple comparisons test). *P* < 0.0001 for *ubc4-1*, *P* = 0.0172 for *bdf2Δ*, *P* = 0.0028 for *ubc4-1 epe1Δ* and *P* < 0.0001 for *ubc4-1 bdf2Δ*.

e and **f** WB analyses for Epe1-Flag and Bdf2-Flag in the indicated strains. Ponceau S staining is used as control. Molecular weight markers are shown and uncropped images are provided in a Source Data file.

g RT-qPCR analysis for *epe1* transcripts in the indicated strains. Data are presented as mean ± SD (n = 2).

h Assays for silencing of centromeric *ura4*⁺ and *ade6*⁺ reporter genes on FOA and Low Ade media in the indicated strains.

i ChIP-seq reads of H3K9me2 (blue) and H3K9me3 (green) mapped to centromere 3 (*cnt3*) right arm in the indicated strains.

j ChIP-qPCR assays for enrichment of H3K9me2 and H3K9me3 at centromeric *dh* repeat in the indicated strains. Data are presented as mean ± SD (n = 2). *P* values are from one-way ANOVA test (Dunnett's multiple comparisons test). *P* = 0.0045 for *cul4-1* and *P* = 0.0287 for *epe1-OE* (left). *P* = 0.0013 for *cul4-1* and *P* = 0.0008 for *epe1-OE* (right).

k Schematic diagram showing the presumptive subunit arrangement in the complex of Ubc4-CLRC and its substrate Bdf2. Poly-ubiquitination activity of Ubc4-CLRC toward Bdf2 promotes degradation of Bdf2, reduced RNA Pol II recruitment to centromeric heterochromatin and subsequently reduced centromeric sRNA production. Red circle (U), ubiquitin.

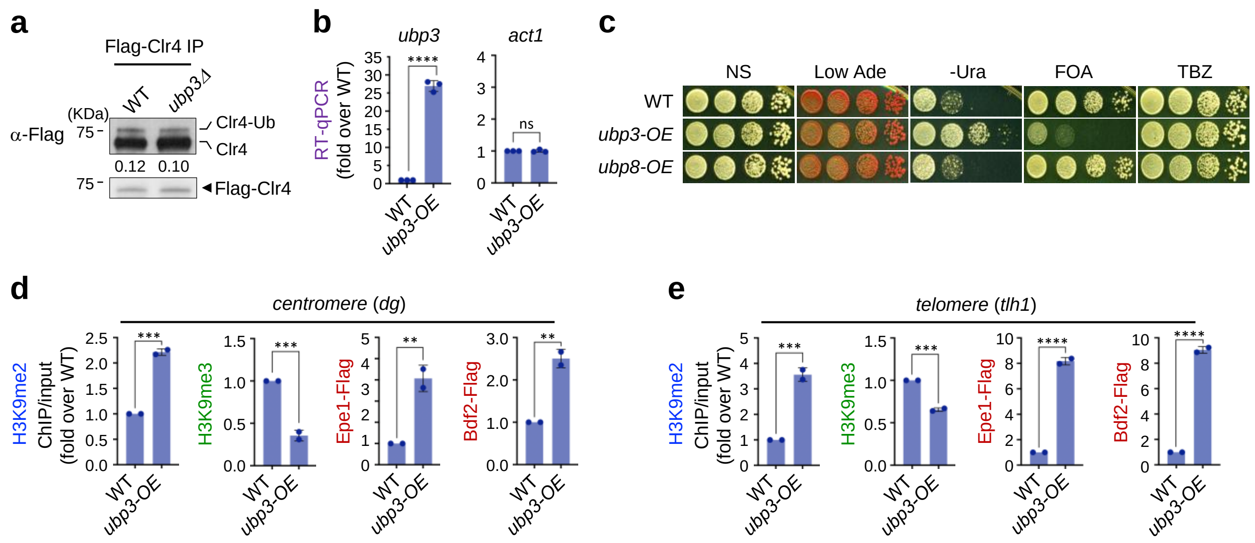

Supplementary Fig. 11│ Ubp3 counteracts Ubc4-CLRC activities.

a WB analyses of Flag-Clr4 IP reveals that *ubp3* deletion does not cause significant change of Clr4 ubiquitination. Molecular weight markers are shown and uncropped images are provided in a Source Data file.

b RT-qPCR analysis for *ubp3*, centromeric *dg* and *act1* transcripts in the indicated strains. Data are presented as mean ± SD (n = 3). *P* values are from one-way ANOVA test (Dunnett's multiple comparisons test). *P* < 0.0001 for *ubp3-OE* (left).

c Assays for silencing of centromeric *ade6*+ and *ura4*+ reporter genes on Low Ade, -Ura and FOA media and for chromosome segregation by viability on 15 µg/ml TBZ medium in *ubp3* or *ubp8* overexpression (-OE) cells.

d ChIP-qPCR assays for enrichment of H3K9me2, H3K9me3, Epe1-Flag and Bdf2-Flag at centromeric *dg* locus. Data are presented as mean ± SD (n = 2). *P* values are from one-way ANOVA test (Dunnett's multiple comparisons test). *P* = 0.0001 for *ubp3-OE* (H3K9me2), *P* = 0.0007 for *ubp3-OE* (H3K9me2), *P* = 0.0056 for *ubp3-OE* (Epe1-Flag) and *P* = 0.0021 for *ubp3-OE* (Bdf2-Flag)

e ChIP-qPCR assays for enrichment of H3K9me2, H3K9me3, Epe1-Flag and Bdf2-Flag at telomeric *tlh1* locus. Data are presented as mean ± SD (n = 2). *P* values are from one-way ANOVA test (Dunnett's multiple comparisons test). *P* = 0.0008 for *ubp3-OE* (H3K9me2), *P* = 0.0002 for *ubp3-OE* (H3K9me2) and *P* < 0.0001 for *ubp3-OE* (Epe1-Flag) and *ubp3-OE* (Bdf2-Flag).

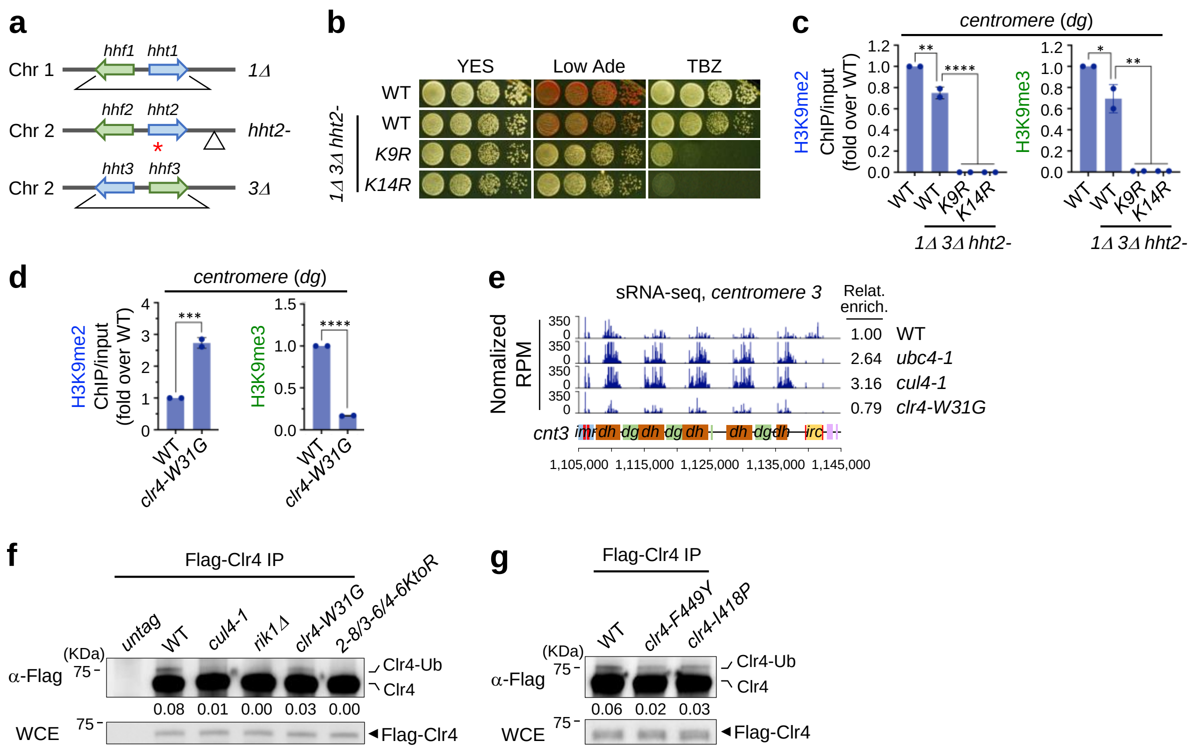

Supplementary Fig. 12 | Mutation of histone H3 lysines 9 and 14 abolishes both H3K9me2 and H3K9me3, while *clr4* mutants with H3K9me2-to-H3K9me3 transition defect show reduced mono-ubiquitination.
a Schematic diagram of histone H3 and H4 genes. After the *hhf1-hht1* and *hht3-hhf3* gene pairs were deleted (open trapezoid, *1Δ 3Δ*), the *hht2* gene was mutagenized by inserting a marker gene (triangle) and simultaneous homologous recombination (*hht2-*). Asterisk indicates mutation site in *hht2*.
b Assays for silencing of centromeric *ade6*⁺ on Low Ade medium and for chromosome segregation by viability on 15 μg/ml TBZ medium in the indicated strains. Both H3K14R and H3K9R mutants have a strong silencing defect with white colonies on Low Ade medium, and high sensitivity to TBZ.
c ChIP-qPCR assays for enrichment of H3K9me2 and H3K9me3 at centromeric *dg* repeats in the indicated strains. Both H3K14R and H3K9R mutants completely lost H3K9me2 and H3K9me3 at the centromere. Data are presented as mean ± SD (n = 2). *P* values are from one-way ANOVA test (Dunnett's multiple comparisons test). *P* = 0.0021 for *1Δ 3Δ hht2*-WT and *P* < 0.0001 for *1Δ 3Δ hht2-K9R and 1Δ 3Δ hht2-K14R* (left). *P* = 0.0238 for *1Δ 3Δ hht2*-WT and *P* = 0.0012 for *1Δ 3Δ hht2-K9R and 1Δ 3Δ hht2-K14R* (right).
d ChIP-qPCR assays showing enrichment of H3K9me2 (left), H3K9me3 (right) at centromeric *dg* repeat in the indicated strains. Data are presented as mean ± SD (n = 2). *P* values are from one-way ANOVA test (Dunnett's multiple comparisons test). *P* = 0.0006 for *clr4-W31G* (left) and *P* < 0.0001 for *clr4-W31G* (right).
e sRNA-seq reads mapped to centromere 3 right arm in indicated strains.
f and **g** WB analyses of Flag-Clr4 IP from indicated strains. Molecular weight markers are shown and uncropped images are provided in a Source Data file.

Table S1. *S. pombe* strains used in this study.

Number	Name	Genotype	Reference
FY2002	WT	*h^+ otrIR(SphI)::ade6 ImrIL(NcoI)::leul-32 ade6-DN/N ura4-DS/E leu1*	R. Allshire
HSK29	*ubc4-1*	FY2002_*ubc4-G48D-KanMX6*	This study
HSK335	*cul4-1*	FY2002_*cul4-GFP-KanMX6*	This study
HSK458	*cul4-1 ubc4-1*	FY2002_*cul4-GFP-KanMX6 ubc4-G48D-NatMX6*	This study
HSK608	*ubc4::mUBE2D2*	FY2002_*KanMX6-ubc4::mUBE2D2*	This study
HSK610	*ubc4::mUBE2D2-1*	FY2002_*KanMX6-ubc4::mUBE2D2-G48D*	This study
HSK351	*clr4Δ*	FY2002_*clr4Δ::KanMx6*	This study
HSK770	*dcr1Δ*	*FY2002_dcr1Δ::NatMx6*	This study
HSK790	*dcr1Δ ubc4-1*	FY2002_*dcr1Δ::NatMx6 ubc4-G48D-KanMX6*	This study
HSK644	*ubc4-3xHA*	FY2002_*ubc4-3xHA-KanMX6*	This study
HSK646	*cul4Δ*	FY2002_*cul4Δ::NatMx6*	This study
HSK679	*GFP-Swi6*	FY2002_*HphMX6-GFP-Swi6*	This study
HSK700	*GFP-Swi6 ubc4-1*	FY2002_*HphMX6-GFP-Swi6 ubc4-G48D-KanMX6*	This study
HSK776	*GFP-Swi6 dcr1Δ*	FY2002_*HphMX6-GFP-Swi6 dcr1Δ::NatMX6*	This study
HSK808	*GFP-Swi6 dcr1Δ ubc4-1*	FY2002_*HphMX6-GFP-Swi6 dcr1Δ::NatMX6 ubc4-G48D-KanMX6*	This study
HSK778	*GFP-Swi6 clr4Δ*	FY2002_*HphMX6-GFP-Swi6 clr4Δ::KanMX6*	This study
HSK716	*GFP-Swi6 ubc4-3xHA*	FY2002_*HphMX6-GFP-Swi6 ubc4-3xHA-KanMX6*	This study
HSK717	*GFP-Swi6 cul4Δ*	FY2002_*HphMX6-GFP-Swi6 cul4Δ::NatMX6*	This study
KFP26	h^-	*h^- ura4-D18 leu1-32 ade6-M210 his3-D1*	Lab stock
SPG18	h^{90}	*h^{90} ura4-D18 leul-32 ade6 -M216 his2*	Lab stock
DI304	*h^{90} ubc4-1*	*h^{90} leul-32 ubc4-G48D-KanMX6*	Irvine, 2009
AY709	*h^{90}ubc4-1/S137T*	*h^{90} leul-32 ubc4-G48D-KanMX6 ubc4-S137T* (EMS mutagenesis)	This study
HSK438	*ubc4-1*	FY2002_*KanMX6-ubc4-G48D*	This study
HSK440	*ubc4-S137T*	FY2002_*ubc4-S137T-HphMX6*	This study
HSK442	*ubc4-1/S137T*	FY2002_*KanMX6-ubc4-G48D/S137T-HphMX6*	This study
HSK603	*swi6Δ*	FY2002_*swi6Δ::KanMx6*	This study
HSK393	*3xFlag-Rik1*	FY2002_*HphMX6-3xFlag-Rik1*	This study
HSK389	*3xFlag-Raf1*	FY2002_*HphMX6-3xFlag-Raf1*	This study
HSK274	*Raf2-3xFlag*	FY2002_*Raf2-3xFlag-HphMX6*	This study
HSK513	*epe1Δ*	FY2002_*epe1Δ::NatMX6*	This study
HSK525	*ubc4-1 epe1Δ*	FY2002_*ubc4-G48D-KanMX6 epe1Δ::NatMX6*	This study
HSK527	*cul4-1 epe1Δ*	FY2002_*cul4-GFP-KanMX6 epe1Δ::NatMX6*	This study
HSK518	*bdf2Δ*	FY2002_*bdf2Δ::KanMX6*	This study
HSK543	*ubc4-1 bdf2Δ*	FY2002_*ubc4-G48D-KanMX6 bdf2Δ::NatMX6*	This study
HSK545	*cul4-1 bdf2Δ*	FY2002_*cul4-GFP-KanMX6 bdf2Δ::NatMX6*	This study
HSK626	*epe1-OE*	FY2002_*KanMX6-adh1p-epe1*	This study
HSK522	*Epe1-3xFlag*	FY2002_ *Epe1-3xFlag-HphMX6*	This study
HSK561	*Epe1-3xFlag ubc4-1*	FY2002_ *Epe1-3xFlag-HphMX6 ubc4-G48D-KanMX6*	This study
HSK563	*Epe1-3xFlag cul4-1*	FY2002_ *Epe1-3xFlag-HphMX6 cul4-GFP-KanMX6*	This study
HSK634	*epe1-OE-3xFlag*	FY2002_*kanMX6-adh1p-epe1-3xFlag-HphMX6*	This study
HSK524	*Bdf2-3xFlag*	FY2002_ *Bdf2-3xFlag-HphMX6*	This study
HSK571	*Bdf2-3xFlag ubc4-1*	FY2002_ *Bdf2-3xFlag-HphMX6 ubc4-G48D-KanMX6*	This study
HSK573	*Bdf2-3xFlag cul4-1*	FY2002_ *Bdf2-3xFlag-HphMX6 cul4-GFP-KanMX6*	This study
HSK1259	*Bdf2-3xFlag epe1-OE*	FY2002_ *Bdf2-3xFlag-HphMX6 KanMX6-adh1p-epe1*	This study
HSK355	*rik1Δ*	FY2002_*rik1Δ::KanMx6*	This study
HSK585	*Epe1-3xFlag ddb1Δ*	FY2002_ *Epe1-3xFlag-HphMX6 ddb1Δ::KanMx6*	This study
HSK565	*Epe1-3xFlag clr3Δ*	FY2002_ *Epe1-3xFlag-HphMX6 clr3Δ::KanMX6*	This study
HSK1097	*Bdf2-3xFlag rik1Δ*	FY2002_ *Bdf2-3xFlag-HphMX6 rik1Δ::KanMx6*	This study
HSK1099	*Bdf2-3xFlag raf1Δ*	FY2002_ *Bdf2-3xFlag-HphMX6 raf1Δ::KanMx6*	This study
HSK1101	*Bdf2-3xFlag raf2Δ*	FY2002_ *Bdf2-3xFlag-HphMX6 raf2Δ::KanMx6*	This study
HSK606	*3xFlag-Clr4*	FY2002_*HphMX6-3xFlag-Clr4*	This study

Number	Name	Genotype	Reference
HSK618	3xFlag-Clr4 ubc4-1	FY2002_HphMX6-3xFlag-Clr4 ubc4-G48D-KanMX6	This study
HSK620	3xFlag-Clr4 cul4-1	FY2002_HphMX6-3xFlag-Clr4 cul4-GFP-KanMX6	This study
HSK920	3xFlag-Clr4 rik1Δ	FY2002_HphMX6-3xFlag-Clr4 rik1Δ::NatMx6	This study
HSK921	3xFlag-Clr4 raf1Δ	FY2002_HphMX6-3xFlag-Clr4 raf1Δ::NatMx6	This study
HSK922	3xFlag-Clr4 raf2Δ	FY2002_HphMX6-3xFlag-Clr4 raf2Δ::NatMx6	This study
HSK1118	3xFlag-Clr4-AllKtoR	FY2002_HphMX6-3xFlag-Clr4-AllKtoR	This study
HSK1125	3xFlag-Clr4-1-6KtoR	FY2002_HphMX6-3xFlag-Clr4-1-6KtoR	This study
HSK971	3xFlag-Clr4-2-8KtoR	FY2002_HphMX6-3xFlag-Clr4-2-8KtoR	This study
HSK1127	3xFlag-Clr4-3-6KtoR	FY2002_HphMX6-3xFlag-Clr4-3-6KtoR	This study
HSK1129	3xFlag-Clr4-4-6KtoR	FY2002_HphMX6-3xFlag-Clr4-4-6KtoR	This study
HSK1131	3xFlag-Clr4-5-5KtoR	FY2002_HphMX6-3xFlag-Clr4-5-5KtoR	This study
HSK1132	3xFlag-Clr4-6-5KtoR	FY2002_HphMX6-3xFlag-Clr4-6-5KtoR	This study
HSK1121	3xFlag-Clr4-2-4KtoR	FY2002_HphMX6-3xFlag-Clr4-2-4KtoR	This study
HSK1140	3xFlag-Clr4-K87R	FY2002_HphMX6-3xFlag-Clr4-K87R	This study
HSK1142	3xFlag-Clr4-K94R	FY2002_HphMX6-3xFlag-Clr4-K94R	This study
HSK1144	3xFlag-Clr4-K109R	FY2002_HphMX6-3xFlag-Clr4-K109R	This study
HSK1146	3xFlag-Clr4-K110R	FY2002_HphMX6-3xFlag-Clr4-K110R	This study
HSK1148	3xFlag-Clr4-K113R	FY2002_HphMX6-3xFlag-Clr4-K113R	This study
HSK1150	3xFlag-Clr4-K114R	FY2002_HphMX6-3xFlag-Clr4-K114R	This study
HSK1152	3xFlag-Clr4-K122R	FY2002_HphMX6-3xFlag-Clr4-K122R	This study
HSK1154	3xFlag-Clr4-K127R	FY2002_HphMX6-3xFlag-Clr4-K127R	This study
HSK1013	3xFlag-clr4-K109/110/113/114R	FY2002_HphMX6-3xFlag-Clr4-K109/110/113/114R-KanMX6	This study
HSK999	3xFlag-Clr4-K87/94/109/110/113/114R	FY2002_HphMX6-3xFlag-Clr4-K87/94/109/110/113/114R-KanMX6	This study
HSK1000	3xFlag-Clr4-K109/110/113/114/122/127R	FY2002_HphMX6-3xFlag-Clr4-K109/110/113/114/122/127R-KanMX6	This study
HSK1015	3xFlag-Clr4-K110/113/114R	FY2002_HphMX6-3xFlag-Clr4-110/113/114R-KanMX6	This study
HSK1017	3xFlag-Clr4-K109/113/114R	FY2002_HphMX6-3xFlag-Clr4-109/113/114R-KanMX6	This study
HSK1019	3xFlag-Clr4-K109/110/114R	FY2002_HphMX6-3xFlag-Clr4-109/110/114R-KanMX6	This study
HSK1021	3xFlag-Clr4-K109/110/113R	FY2002_HphMX6-3xFlag-Clr4-109/110/113R-KanMX6	This study
HSK1082	3xFlag-Clr4-K109/110R	FY2002_HphMX6-3xFlag-Clr4-109/110R-KanMX6	This study
HSK1062	3xFlag-Clr4-K109/113R	FY2002_HphMX6-3xFlag-Clr4-109/113R-KanMX6	This study
HSK1064	3xFlag-Clr4-K109/114R	FY2002_HphMX6-3xFlag-Clr4-109/114R-KanMX6	This study
HSK1086	3xFlag-Clr4-K110/113R	FY2002_HphMX6-3xFlag-Clr4-110/113R-KanMX6	This study
HSK1065	3xFlag-Clr4-K110/114R	FY2002_HphMX6-3xFlag-Clr4-110/114R-KanMX6	This study
HSK1084	3xFlag-Clr4-K113/114R	FY2002_HphMX6-3xFlag-Clr4-113/114R-KanMX6	This study
HSK967	clr4-2-8KtoR	FY2002_HphMX6-clr4-2-8KtoR	This study
HSK1124	clr4-2-4KtoR	FY2002_HphMX6-clr4-2-4KtoR	This study
HSK1080	clr4-W31G	FY2002_HphMX6-clr4-W31G	This study
HSK1049	3xFlag-Clr4-W31G	FY2002_HphMX6-3xFlag-Clr4-W31G	This study
HSK1054	3xFlag-Clr4-W31G ubc4-1	FY2002_HphMX6-3xFlag-Clr4-W31G ubc4-G48D-KanMX6	This study
HSK927	3xFlag-Clr4-I418P	FY2002_HphMX6-3xFlag-Clr4-I418P	This study
HSK928	3xFlag-Clr4-F449Y	FY2002_HphMX6-3xFlag-Clr4-F449Y	This study
HSK1299	5xUAS-Ade6	HSK1290 ($h^?$ ade6-DN/N $ura4^+$)_ura4::5xUAS-ade6	This study
HSK1313	5xUAS-Ade6 GBD-Clr4-ΔCD Clr4	HSK1299_GBD-Clr4-ΔCD leu1::Clr4	This study
HSK1319	5xUAS-Ade6 GBD-Clr4-ΔCD Clr4 ubc4-1	HSK1313_ubc4-G48D-KanMX6	This study
HSK1321	5xUAS-Ade6 GBD-Clr4-ΔCD Clr4 cul4-1	HSK1313_cul4-GFP-KanMX6	This study
HSK395	ubp3-OE	FY2002_HphMX6-adh1p-ubp3	This study
HSK401	ubp8-OE	FY2002_HphMX6-adh1p-ubp8	This study
HSK918	3xFlag-Clr4 ubp3-OE	FY2002_HphMX6-3xFlag-Clr4 NatMX6-adh1p-ubp3	This study
HSK1277	Epe1-3xFlag ubp3-OE	FY2002_ Epe1-3xFlag-HphMX6 NatMX6-adh1p-ubp3	This study
HSK1279	Bdf2-3xFlag ubp3-OE	FY2002_ Bdf2-3xFlag-HphMX6 NatMX6-adh1p-ubp3	This study
HSK919	3xFlag-Clr4 ubp3Δ	FY2002_HphMX6-3xFlag-Clr4 ubp3Δ::NatMx6	This study
HSK388	1Δ 3Δ WT	FY2002_hhf1-hht1Δ::HphMX6 hht3-hhf3Δ::NatMX6	This study
HSK480	1Δ 3Δ hht2-K9R	HSK388_ hht2-K9R-KanMX6	This study
HSK482	1Δ 3Δ hht2-K14R	HSK388_ hht2-K14R-KanMX6	This study

Table S2. Plasmids used in this study.

Name	Description	Application
pET-14b-Clr4	6xHis-Clr4	Recombinant 6xHis-Clr4 protein purification from *E. coli*
pHis-parellel-GFP-Clr4^{Full}	6xHis-GFP-Clr4^{Full}	Recombinant 6xHis-GFP-Clr4^{Full} protein purification from *E. coli*
pHis-parellel-mCherry-Clr4^{Full}	6xHis-mCherry-Clr4^{Full}	Recombinant 6xHis-mCherry-Clr4^{Full} protein purification from *E. coli*
pHis-parellel-GFP-Clr4^{IDR}	6xHis-GFP-Clr4^{IDR}	Recombinant 6xHis-GFP-Clr4^{IDR} protein purification from *E. coli*
pET-14b-Flag-Clr4	6xHis-1xFlag-Clr4	Recombinant 6xHis-1xFlag-Clr4 protein purification from *E. coli*
pET-14b-Myc-Clr4	6xHis-1xMyc-Clr4	Recombinant 6xHis-1xMyc-Clr4 protein purification from *E. coli*
pET-14b-H3N-GST	H3N-GST-6xHis	Recombinant H3N-GST-6xHis protein purification from *E. coli*

Table S3. Oligonucleotides used in this study.

Name	Description	Purpose	Sequence
p30-qPCR-F	*dg*	ChIP-qPCR, RT-qPCR	CCATATCAATTTCCCATGTTCC
p30-qPCR-R	*dg*	ChIP-qPCR, RT-qPCR	CATCAAGCGAGTCGAGATGA
p33-qPCR-F	*dh*	ChIP-qPCR, RT-qPCR	TATCCTGCGTCTCGGTATCC
p33-qPCR-R	*dh*	ChIP-qPCR, RT-qPCR	CTGTTCGTGAATGCTGAGAAAG
act1-qPCR-F	*act1*	ChIP-qPCR, RT-qPCR	TGCACCTGCCTTTTATGTTG
act1-qPCR-F	*act1*	ChIP-qPCR, RT-qPCR	TGGGAACAGTGTGGGTAACA
ade6-qPCR-F	*ade6*	ChIP-qPCR	TTCCTACTGCCATCAAAGCA
ade6-qPCR-R	*ade6*	ChIP-qPCR	GCACGCTGTTGAATTGAGAA
ubp3-qPCR-F	*ubp3*	RT-qPCR	GCCAAATTTTTGGTGGTCAG
ubp3-qPCR-R	*ubp3*	RT-qPCR	TGAATGTCGAGTTGCAGAGG
epe1-qPCR-F	*epe1*	RT-qPCR	GCAATTCTTCAAATCCATCCA
epe1-qPCR-R	*epe1*	RT-qPCR	TCGAGTCGTGATGGAATTGA

Table S4. Antibodies used in this study.

Name	Company & Catalogue Numer	Description	Application
α-UBE2D3	Proteintech 11677-1-AP	Rabbit polyclonal	WB (Western Blot)
α-RNA Pol II	BioLegend 904001	Mouse monoclonal	ChIP (Chromatin immunoprecipitation)
α-H3K9me2	Abcam, ab1220	Mouse monoclonal	ChIP and WB
α-H3K9me3	Absolute Antibody Ab00700-1.26	Mouse scFv-Fc (synthetic antibody)	ChIP and WB
α-H3K9me3	Abcam ab8898	Rabbit polyclonal	WB
α-H3	Abcam ab1791	Rabbit polyclonal	WB
α-GFP	Sigma 11814460001 (Roche)	mouse monoclonal	IP (immunoprecipitation)
α-GFP	Abcam ab290	Rabbit polyclonal	WB
α-Flag Agarose	Sigma A2220	Mouse monoclonal	IP
α-Flag	Sigma F7425	Rabbit polyclonal	WB
α-Flag	Sigma F1804	Mouse monoclonal	ChIP and RNA IP
α-Myc	Abcam ab9106	Rabbit polyclonal	WB
α-His	ThermoFisher MA1-135	Mouse monoclonal	WB
α-HA	Roche 11583816001	Mouse monoclonal	WB
α-Swi6	Abcam ab188276	Rabbit polyclonal	ChIP
α-Ago1	Abcam Ab18190	Rabbit polyclonal	ChIP
α-Chp1	Abcam ab18191	Rabbit polyclonal	ChIP
α-Tubulin	Abcam ab6160	Rabbit monoclonal	WB
α-Ubiquitin	Enzo Life Sciences #ADI-SPA-200-F	Rabbit Polyclonal	WB
UBA TUBE	Cytoskeleton # UBA01-beads	Signal-Seeker Ubiquitination Affinity Beads	UBA TUBE IP
