# Supplementary material for: Clr4SUV39H1 ubiquitination and non-coding RNA mediate transcriptional silencing of heterochromatin via Swi6 phase separation
Source: Nat Commun. 2024 Oct 30;15:9384. doi: 10.1038/s41467-024-53417-9 (PMC11526040; doi:10.1038/s41467-024-53417-9)
Supplement: Supplementary file 3 — Reporting Summary [file 41467_2024_53417_MOESM3_ESM.pdf]

nature portfolio

Corresponding author(s): Robert A. Martienssen

Last updated by author(s): Dec 4, 2023

Reporting Summary

Nature Portfolio wishes to improve the reproducibility of the work that we publish. This form provides structure for consistency and transparency in reporting. For further information on Nature Portfolio policies, see our Editorial Policies and the Editorial Policy Checklist.

Statistics

For all statistical analyses, confirm that the following items are present in the figure legend, table legend, main text, or Methods section.

n/a	Confirmed	
☐	☒	The exact sample size (*n*) for each experimental group/condition, given as a discrete number and unit of measurement
☐	☒	A statement on whether measurements were taken from distinct samples or whether the same sample was measured repeatedly
☐	☒	The statistical test(s) used AND whether they are one- or two-sided<br>*Only common tests should be described solely by name; describe more complex techniques in the Methods section.*
☒	☐	A description of all covariates tested
☒	☐	A description of any assumptions or corrections, such as tests of normality and adjustment for multiple comparisons
☐	☒	A full description of the statistical parameters including central tendency (e.g. means) or other basic estimates (e.g. regression coefficient) AND variation (e.g. standard deviation) or associated estimates of uncertainty (e.g. confidence intervals)
☐	☒	For null hypothesis testing, the test statistic (e.g. *F*, *t*, *r*) with confidence intervals, effect sizes, degrees of freedom and *P* value noted<br>*Give P values as exact values whenever suitable.*
☒	☐	For Bayesian analysis, information on the choice of priors and Markov chain Monte Carlo settings
☒	☐	For hierarchical and complex designs, identification of the appropriate level for tests and full reporting of outcomes
☒	☐	Estimates of effect sizes (e.g. Cohen's *d*, Pearson's *r*), indicating how they were calculated

Our web collection on statistics for biologists contains articles on many of the points above.

Software and code

Policy information about availability of computer code

Data collection	No software was used for data collection in this study.
Data analysis	Raw reads of ChIP-seq were trimmed using Trimmomatic, mapped to S. pombe genome using Bowtie2 and visualized in IGV genome browser. Total ChIP read counts for defined region were analyzed using MultiBigwigSummary or MultiBamSummary. Raw reads of sRNA-seq were trimmed using FASTX-Toolkit, keeping a minimum read size of 15 nt, and mapped to the S. pombe genome using Bowtie allowing up to 1 mismatch. The strand-specific genomic coverage was calculated using Bedtools41 and represented in 20 nt windows.

For manuscripts utilizing custom algorithms or software that are central to the research but not yet described in published literature, software must be made available to editors and reviewers. We strongly encourage code deposition in a community repository (e.g. GitHub). See the Nature Portfolio guidelines for submitting code & software for further information.

Data

Policy information about availability of data

All manuscripts must include a data availability statement. This statement should provide the following information, where applicable:

- Accession codes, unique identifiers, or web links for publicly available datasets
- A description of any restrictions on data availability
- For clinical datasets or third party data, please ensure that the statement adheres to our policy

All raw and processed files from Next-Generaton Sequencing experiments are available at GEO with accession number GSE156069.

Research involving human participants, their data, or biological material

Policy information about studies with human participants or human data. See also policy information about sex, gender (identity/presentation), and sexual orientation and race, ethnicity and racism.

Reporting on sex and gender	Not applicable to this study.
Reporting on race, ethnicity, or other socially relevant groupings	Not applicable to this study.
Population characteristics	Not applicable to this study.
Recruitment	Not applicable to this study.
Ethics oversight	Not applicable to this study.

Note that full information on the approval of the study protocol must also be provided in the manuscript.

Field-specific reporting

Please select the one below that is the best fit for your research. If you are not sure, read the appropriate sections before making your selection.

☒ Life sciences ☐ Behavioural & social sciences ☐ Ecological, evolutionary & environmental sciences

For a reference copy of the document with all sections, see nature.com/documents/nr-reporting-summary-flat.pdf

Life sciences study design

All studies must disclose on these points even when the disclosure is negative.

Sample size	At least 2 or 3 times of biological replicates were tested to calculate mean and Standard Deviation (S.D.).
Data exclusions	No data were excluded.
Replication	All experiments were repeated with independent biological replicates prepared from different sources of culture. The precise number of biological replicates are provided in the figure legend. ChIP-qPCR and RT-qPCR experiments were tested with 2 or 3 independent biological replicates. ChIP-seq and sRNA-seq experiments were tested with at least 2 biological replicates. Silencing assays on agar plates were repeated at least 2 times on different days with similar results. Western blot assays after immunoprecipitation were tested more than 2 times with similar results. Mass spectrometry results were tested at least 3 times to optimize the condition. For microscopy, minimum 10 cells were tested in each condition. In vitro Liquid-Liquid Phase Separation (LLPS) assays were tested more than 3 times with similar results.
Randomization	Not applicable to this study.
Blinding	Not applicable to this study.

Reporting for specific materials, systems and methods

We require information from authors about some types of materials, experimental systems and methods used in many studies. Here, indicate whether each material, system or method listed is relevant to your study. If you are not sure if a list item applies to your research, read the appropriate section before selecting a response.

Materials & experimental systems

n/a	Involved in the study	
☐	☒	Antibodies
☒	☐	Eukaryotic cell lines
☒	☐	Palaeontology and archaeology
☒	☐	Animals and other organisms
☒	☐	Clinical data
☒	☐	Dual use research of concern
☒	☐	Plants

Methods

n/a	Involved in the study	
☐	☒	ChIP-seq
☒	☐	Flow cytometry
☒	☐	MRI-based neuroimaging

Antibodies

Antibodies used

Anti-H3 (Abcam ab1791), anti-H3K9me2 (Abcam, ab1220), anti-H3K9me3 (Absolute Antibody Ab00700-1.26, for ChIP), anti-H3K9me3 (ab8898, for Western blot), anti-RNA Pol II (BioLegend 904001), anti-Swi6 (Abcam ab188276), anti-Ago1 (Abcam Ab18190), anti-Chp1 (Abcam ab18191), anti-Flag (Sigma F1804, for ChIP and Western blot), anti-Flag (Sigma F7425, for Western blot), anti-Flag agarose (Sigma A2220, for Immunoprecipitation), anti-HA (Roche 11583816001), anti-His (ThermoFisher MA1-135), anti-mUBE2D3 (Proteintech 11677-1-AP), anti-ubiquitin (Enzo Life Sciences #ADI-SPA-200-F), UBA beads (Cytoskeleton # UBA01-beads), anti-GFP (Sigma 11814460001, for Immunoprecipitation), anti-GFP (Abcam ab290, for Western blot) and anti-Tubulin (Abcam ab6160).

Validation

Negative controls like untagged strain or deletion mutant were used to validate antibodies.

Plants

Seed stocks

Not applicable to this study.

Novel plant genotypes

Not applicable to this study.

Authentication

Not applicable to this study.

ChIP-seq

Data deposition

☒ Confirm that both raw and final processed data have been deposited in a public database such as GEO.

☐ Confirm that you have deposited or provided access to graph files (e.g. BED files) for the called peaks.

Data access links
May remain private before publication.

https://www.ncbi.nlm.nih.gov/geo/query/acc.cgi?acc=GSE156069
Access token: qzgzkcwmzrafrgz

Files in database submission

GSM6409915 01-01_H3K9me2 ChIP-seq_WT
GSM6409917 01-02_H3K9me2 ChIP-seq_ubc4-1
GSM6409918 01-03_H3K9me2 ChIP-seq_cul4-1
GSM6409919 01-04_H3K9me2 ChIP-seq_dcr1
GSM6409920 01-05_H3K9me2 ChIP-seq_dcr1 ubc4-1
GSM6409921 01-06_H3K9me2 ChIP-seq_clr4
GSM6409922 01-07_H3K9me3 ChIP-seq_WT
GSM6409924 01-08_H3K9me3 ChIP-seq_ubc4-1
GSM6409925 01-09_H3K9me3 ChIP-seq_cul4-1
GSM6409926 01-10_H3K9me3 ChIP-seq_dcr1
GSM6409927 01-11_H3K9me3 ChIP-seq_dcr1 ubc4-1
GSM6409928 01-12_H3K9me3 ChIP-seq_clr4
GSM6409929 01-13_Rpb1 ChIP-seq_WT
GSM6409931 01-14_Rpb1 ChIP-seq_ubc4-1
GSM6409932 01-15_Rpb1 ChIP-seq_cul4-1
GSM6409933 01-16_Rpb1 ChIP-seq_dcr1
GSM6409934 01-17_Rpb1 ChIP-seq_dcr1 ubc4-1
GSM6409935 01-18_Rpb1 ChIP-seq_clr4
GSM6409936 02-01_Epe1-Flag_untag
GSM6409937 02-02_Epe1-Flag_WT
GSM6409939 02-03_Epe1-Flag_ubc4-1
GSM6409940 02-04_Epe1-Flag_cul4-1
GSM6409941 02-05_Epe1-Flag_epe1-OE
GSM6409942 02-06_Bdf2-Flag_untag
GSM6409943 02-07_Bdf2-Flag_WT
GSM6409944 02-08_Bdf2-Flag_ubc4-1
GSM6409945 02-09_Bdf2-Flag_cul4-1
GSM6409947 02-10_Bdf2-Flag_epe1-OE
GSM6409948 03-01_H3K9me2 ChIP-seq_WT
GSM6409949 03-02_H3K9me2 ChIP-seq_cul4-1
GSM6409950 03-03_H3K9me2 ChIP-seq_epe1-OE
GSM6409951 03-04_H3K9me3 ChIP-seq_WT
GSM6409952 03-05_H3K9me3 ChIP-seq_cul4-1
GSM6409954 03-06_H3K9me3 ChIP-seq_epe1-OE
GSM6409955 04-01_H3K9me2 ChIP-seq_WT

GSM6409956 04-02_H3K9me2 ChIP-seq_clr4-2-8KtoR
GSM6409957 04-03_H3K9me2 ChIP-seq_clr4-2-4KtoR
GSM6409958 04-04_H3K9me2 ChIP-seq_clr4-AllKtoR
GSM6409959 04-05_H3K9me3 ChIP-seq_WT
GSM6409961 04-06_H3K9me3 ChIP-seq_clr4-2-8KtoR
GSM6409962 04-07_H3K9me3 ChIP-seq_clr4-2-4KtoR
GSM6409963 04-08_H3K9me3 ChIP-seq_clr4-AllKtoR
GSM6409964 05-01_Flag-Clr4 ChIP-seq_untag
GSM6409965 05-02_Flag-Clr4 ChIP-seq_WT
GSM6409966 05-03_Flag-Clr4 ChIP-seq_ubc4-1
GSM6409968 05-04_Flag-Clr4 ChIP-seq_cul4-1
GSM6409969 05-05_Flag-Clr4 ChIP-seq_Clr4-W31G
GSM6409970 05-06_Flag-Clr4 ChIP-seq_Clr4-W31G ubc4-1
GSM6409971 05-07_Flag-Clr4 ChIP-seq_Clr4-AllKtoR
GSM6409972 06-01_H3K9me2 ChIP-seq_WT
GSM6409973 06-02_H3K9me2 ChIP-seq_ubp3-OE
GSM6409975 06-03_H3K9me3 ChIP-seq_WT
GSM6409976 06-04_H3K9me3 ChIP-seq_ubp3-OE
GSM6409977 06-05_Epe1-Flag ChIP-seq_WT
GSM6409978 06-06_Epe1-Flag ChIP-seq_ubp3-OE
GSM6409979 06-07_Bdf2-Flag ChIP-seq_WT
GSM6409980 06-08_Bdf2-Flag ChIP-seq_ubp3-OE
GSM6409981 07-01_sRNA-seq_WT
GSM6409983 07-02_sRNA-seq_dcr1
GSM6409984 07-03_sRNA-seq_ubc4-1
GSM6409985 07-04_sRNA-seq_cul4-1
GSM6409986 07-05_sRNA-seq_epe1
GSM6409987 07-06_sRNA-seq_ubc4-1 epe1
GSM6409988 07-07_sRNA-seq_cul4-1 epe1
GSM6409990 07-08_sRNA-seq_bdf2
GSM6409991 07-09_sRNA-seq_ubc4-1 bdf2
GSM6409992 07-10_sRNA-seq_cul4-1 bdf2
GSM6409993 07-11_sRNA-seq_epe1-OE
GSM6409994 08-01_sRNA-seq_WT
GSM6409995 08-02_sRNA-seq_ubc4-1
GSM6409996 08-03_sRNA-seq_cul4-1
GSM6409998 08-04_sRNA-seq_clr4-2-8KtoR
GSM6409999 08-05_sRNA-seq_clr4-2-4KtoR
GSM6410000 08-06_sRNA-seq_clr4-W31G
GSM6410001 09-01_sRNA-seq_WT
GSM6410002 09-02_sRNA-seq_ubp3-OE

Genome browser session
(e.g. UCSC)

Not applicable to this study. ChIP-seq results were visualized using IGV genome browser.

Methodology

Replicates	ChIP-seq experiments were tested with at least 2 biological replicates.
Sequencing depth	All ChIP-seq libraries were sequenced with paired-end 101 bp reads using Illumina MiSeq platform. Each samples have more than 1 million reads in normal condition like wild type strain. Uniquely mapped reads are not calculated in this study.
Antibodies	anti-H3K9me2 (Abcam, ab1220), anti-H3K9me3 (Absolute Antibody Ab00700-1.26), and anti-Flag (Sigma F1804).
Peak calling parameters	Specific loci of interest were visualized and the number of reads were calculated without peak calling.
Data quality	ChIP-seq quality was confirmed by lack of peaks in negative control like Clr4 deletion mutant which completely lost H3K9me2/3 ChIP-seq enrichment.
Software	Raw reads of ChIP-seq were trimmed using Trimmomatic, mapped to S. pombe genome using Bowtie2 and visualized in IGV genome browser. Total ChIP read counts for defined region were analyzed using MultiBigwigSummary or MultiBamSummary.
